# Detection of viable but non-culturable *Pseudomonas aeruginosa* in cystic fibrosis by qPCR: a validation study

**DOI:** 10.1186/s12879-018-3612-9

**Published:** 2018-12-27

**Authors:** Gianmarco Mangiaterra, Mehdi Amiri, Andrea Di Cesare, Sonia Pasquaroli, Esther Manso, Natalia Cirilli, Barbara Citterio, Carla Vignaroli, Francesca Biavasco

**Affiliations:** 1 Department of Life and Environmental Sciences, Polytechnic University of Marche, via Brecce Bianche, 60131 Ancona, Italy; 2 Department of Earth, Environmental and Life Sciences, University of Genoa, Corso Europa, 26, 16132 Genoa, Italy; 3 Microbiology Laboratory, Azienda Ospedaliero-Universitaria, Ospedali Riuniti Umberto I°- G.M. Lancisi - G. Salesi, Ancona, Italy; 4 Mother-Child Department, Cystic Fibrosis Referral Care Center, United Hospitals, Ancona, Italy; 5 Department of Biomolecular Sciences sect. Biotechnology, University of Urbino “Carlo Bo”, Urbino, Italy

**Keywords:** *Pseudomonas aeruginosa*, Viable but non-culturable bacterial forms, qPCR, Cystic fibrosis, Lung infection

## Abstract

**Background:**

Routine culture-based diagnosis of *Pseudomonas aeruginosa* lung infection in Cystic Fibrosis (CF) patients can be hampered by the phenotypic variability of the microorganism, including its transition to a Viable But Non-Culturable (VBNC) state. The aim of this study was to validate an *ecfX-*targeting qPCR protocol developed to detect all viable *P. aeruginosa* bacteria and to identify VBNC forms in CF sputum samples.

**Methods:**

The study involved 115 *P. aeruginosa* strains of different origins and 10 non-*P. aeruginosa* strains and 88 CF sputum samples, 41 Culture-Positive (CP) and 47 Culture-Negative (CN). Spiking assays were performed using scalar dilutions of a mixture of live and dead *P. aeruginosa* ATCC 9027 and a pooled *P. aeruginosa*-free sputum batch. Total DNA from sputum samples was extracted by a commercial kit, whereas a crude extract was obtained from the broth cultures. Extracellular DNA (eDNA) interference was evaluated by comparing the qPCR counts obtained from DNase-treated and untreated aliquots of the same samples. The statistical significance of the results was assessed by the Wilcoxon test and Student’s t test.

**Results:**

The newly-developed qPCR protocol identified 96.6% of the *P. aeruginosa* isolates; no amplification was obtained with strains belonging to different species. Spiking assays supported protocol reliability, since counts always matched the amount of live bacteria, thus excluding the interference of dead cells and eDNA. The protocol sensitivity threshold was 70 cells/ml of the original sample. Moreover, qPCR detected *P. aeruginosa* in 9/47 CN samples and showed higher bacterial counts compared with the culture method in 10/41 CP samples.

**Conclusions:**

Our findings demonstrate the reliability of the newly-developed qPCR protocol and further highlight the need for harnessing a non-culture approach to achieve an accurate microbiological diagnosis of *P. aeruginosa* CF lung infection and a greater understanding of its evolution.

**Electronic supplementary material:**

The online version of this article (10.1186/s12879-018-3612-9) contains supplementary material, which is available to authorized users.

## Background

*Pseudomonas aeruginosa* lung infection represents the main risk of morbidity and mortality in Cystic Fibrosis (CF) patients [1]. After lung colonisation, a wide range of strategies – including biofilm development [2], hypermutation, multidrug resistance, metabolic changes [3], and atypical phenotypes including mucoid forms, small colony variants and slow-growing and even Viable But Non-Culturable (VBNC) forms [4, 5] – promotes pathogen survival and infection persistence. Routine culture-based diagnosis of *P. aeruginosa* lung infection may fail to detect these bacterial populations in CF patients, providing false negative results or underestimating bacterial abundance [5]. A variety of alternative detection approaches have been described [6]. At present, real-time PCR is considered as the best technique to replace culture, and several protocols targeting different species–specific genes have been developed [7–10]. However, most of them seem to possess limited sensitivity and ability to discriminate live from dead cells or extracellular DNA (eDNA) [11]. In this study, we report the diagnostic performance of a newly-developed qPCR protocol targeting *ecfX*, which proved capable of detecting all viable *P. aeruginosa* subpopulations, including VBNC forms, and showed the specificity, sensitivity, ease of use, and cost-effectiveness required for routine microbiological use.

## Methods

### Sample collection and bacterial strains

A total number of 88 sputum samples – 41 Culture-Positive (CP) and 47 Culture-Negative (CN) – from CF patients were randomly and anonymously collected from September 2014 to May 2016 among those sent by the Marche regional Cystic Fibrosis Centre to the Microbiology lab of “Ospedali Riuniti” Hospital (Ancona, Italy). Samples were supplemented 1:1 with Sputasol (Oxoid, Basingstoke, UK) before processing. The study also included 115 *P. aeruginosa* isolates of different origins (51 clinical CF, 55 clinical non-CF, and 9 environmental); 9 recently isolated strains of different bacterial species (1 *Klebsiella pneumoniae*, 1 *Proteus mirabilis* 1 *Achromobacter xylosoxidans,* 1 *Burkholderia cepacia,* 2 *Stenotrophomonas maltophilia,* 2 *Serratia marcescens* and 1 *Morganella morganii*) provided the Microbiology labs of “Ospedali Riuniti” (Ancona, Italy), “Ospedale A. Murri” (Fermo, Italy), and “Arpam” (Pesaro, Italy); and *Escherichia coli* ATCC 25922. *P. aeruginosa* ATCC 9027 was the reference strain.

### Culture-based detection of *P. aeruginosa* in sputum samples

Routine culture assays of CF sputum were performed according to the guidelines described by Gilligan [12] and reported in the UK Consensus Document [13]. *P. aeruginosa* abundance (CFU/ml) was reported as falling in one of the following ranges: < 10^2^ (CN), 10^2^–10^3^; 10^3^–10^4^; 10^4^–10^5^; 10^5^–10^6^.

The 51 clinical CF *P. aeruginosa* isolates were identified by MALDI TOF (VITEK-MS, BioMèrieux, Marcy-l’Étoile, France), whereas the non-CF clinical and environmental strains and the non-*P. aeruginosa* Gram-negative isolates were identified by different systems (MiscroScan Walk Away 40, Beckman Coulter, CA, USA and API 20NE, BioMérieux).

### Spiking assays

Spiking assays were performed by inoculating 10-fold (from 3 × 10^6^ to 3 × 10^2^ cells/ml) or 2-fold (from 1.5 × 10^2^ to 16 cells/ml) scalar dilutions of a mid-log phase culture or of a 1:10 mixture of live (log phase) and dead cells (heat-killed at 75 °C for 15 min) of *P. aeruginosa* ATCC 9027 in a 1 ml aliquot of a pooled sputum batch that was negative for *P. aeruginosa* both by culture and by real-time PCR.

### DNase I digestion

One ml of each sample (sputum or broth culture) was centrifuged at 15,000×g at 4 °C for 15 min, resuspended in 500 μl Phosphate Buffered Saline (PBS), and digested with 18 U DNase I (Ambion-Thermofisher, Carlsbad, CA, USA) at 37 °C for 30 min. After enzyme inactivation at 75 °C for 10 min, samples were washed in 1 ml PBS and resuspended in 100 μl of the same buffer.

### DNA extraction

DNA was extracted from sputum samples using the QIAamp DNA Mini Kit (Qiagen GmbH, Hilden, Germany) according to the manufacturer’s instructions, using 80 μl of elution solution to obtain a DNA concentration between 10 and 10^2^ ng/μl.

DNA amount and purity were verified using an ND-1000 Nanodrop spectrophotometer (Thermo Scientific, Wilmington, NC, USA) and agarose gel electrophoresis. DNA samples were stored at − 20 °C until use.

DNA extraction from broth cultures was performed by a rough extraction protocol as previously described [14].

### PCR assays

The primer combination *ecfX*-F 5’-AGCGTTCGTCCTGCACAAGT-3′ [15] and *ecfX*-R 5’-TCATCCTTCGCCTCCCTG-3′ [7] (amplicon size 145 bp) and the reference strain *P. aeruginosa* ATCC 9027 were used.

Classic PCRs were performed with 5 μl of DNA, 0.5 μM of each primer, and Dream-Taq Polymerase (Thermo Fisher Scientific, Waltham, MA, USA). Amplification conditions were as follows: 10 min at 95 °C, followed by 35 cycles of 30 s at 94 °C, 30 s at 61 °C, 45 s at 72 °C, and a final elongation step of 5 min at 72 °C.

Real-time PCR assays were performed using 0.2 μM of each primer, 10 μl of 2 × Rotor-Gene SYBR Green PCR master mix (Qiagen), and 2 μl DNA. Cycling conditions were 95 °C for 5 min, followed by 33 cycles of 95 °C for 10 s, 61 °C for 30 s, and 72 °C for 20 s. A melting curve was obtained by ramping the temperature from 59 °C to 95 °C (0.5 °C/10 s) and analysed with Qiagen’s Rotor-Gene Q MDx software. Each reaction was run in triplicate. *P. aeruginosa* ATCC 9027 DNA and RNase-free water were the positive and negative control, respectively. The amplicons obtained from *P. aeruginosa* ATCC 9027 and a CF sputum sample were purified with the Gene Elute PCR Clean-up kit (Sigma-Aldrich, Saint Louis, MO, USA) and sequenced bidirectionally (BMR Genomics service (www.bmr-genomics.it/), to confirm their identity. To quantify *P. aeruginosa* cells, a calibration curve was constructed using scalar dilutions (from 10^− 5^ to 10^− 9^ ng/reaction) of an *ecfX* amplicon of *P. aeruginosa* ATCC 9027 DNA; intra- and interassay Coefficients of Variation (CV) were calculated as described previously [16]. Data were analysed using Qiagen’s Rotor Gene Q Series software. *P. aeruginosa* abundance was calculated as reported previously [16] based on amplicon size (145 bp), the weight of 1 bp (1.095 × 10^− 12^ ng), and the number of *ecfX* copies found in the *P. aeruginosa* genome (*n* = 19). The value thus obtained was then doubled considering the 1:1 sample dilution with Sputasol. qPCR and CFU counts differing by < 0.5 log were considered similar.

The Limit Of Detection (LOD) of the qPCR protocol was determined as described previously [17].

### Statistical analysis

The Wilcoxon test and Student’s t test were used for statistical analyses. A *p* value < 0.05 was considered significant.

## Results

### Primer pair sensitivity and specificity

The primer pair targeting *ecfX* was tested by standard PCR assays on 115 *P. aeruginosa* strains of different origins. It produced a correct amplicon in 111/115 (96.6%) isolates; no amplicon was obtained from strains belonging to species other than *P. aeruginosa*. The inability of the protocol to detect 4/115 *P. aeruginosa* strains was not statistically significant (*p* > 0.1), demonstrating that the target sequence is largely conserved (Table 1).

**Table 1:** Amplification of *P. aeruginosa* and non*-P. aeruginosa* strains by the selected *ecfX*-targeting primer pair

Species (no. of strains)	PCR+	PCR-
*P. aeruginosa* - clinical CF (51)	48	3
*P. aeruginosa* - clinical non-CF (55)	54	1
*P. aeruginosa* - environmental (9)	9	0
*Serratia marcescens* (2)	0	2
*Stenotrophomonas maltophilia* (2)	0	2
*Burkholderia cepacia* (1)	0	1
*Achromobacter xylosoxydans* (1)	0	1
*Klebsiella pneumoniae* (1)	0	1
*Morganella morganii* (1)	0	1
*Proteus mirabilis* (1)	0	1
*Escherichia coli* (1)	0	1

### Validation of the real-time PCR protocol

The reliability of the real-time PCR protocol was first assessed by testing for the presence of inhibitors in direct DNA extracts from sputum and by measuring its sensitivity threshold.

#### Purity of DNA extracts

Ten-fold and 100-fold dilutions of the DNA extracts obtained from spiked samples were used to evaluate the presence of PCR inhibitors. The progressive threshold cycle (Ct) average delay of 3.3 cycles for each 10-fold dilution excluded their recovery [18].

#### Accuracy and sensitivity of real-time PCR *P. aeruginosa* direct detection

The accuracy and sensitivity of the real-time PCR protocol were determined by spiking assays using serial dilutions of *P. aeruginosa* ATCC 9027. The melting peaks were always similar to that of a DNA extract from a broth culture of *P. aeruginosa* ATCC 9027. The sensitivity threshold was 70 cells/ml.

The calibration curve obtained from a purified *ecfX* amplicon of *P. aeruginosa* ATCC 9027 yielded regression coefficients close to 1 (R2: 0.998); mean qPCR efficiency was 0.97 ± 0.02. The intra- and interassay CV are reported in Table 2. The LOD was 5.2 × 10^− 9^ ng/reaction, corresponding to about 138 cells/ml of the original sample. The similarity of the qPCR counts and the amount of *P. aeruginosa* cells used for spiking demonstrated that the qPCR protocol reliably detected the microorganism in sputum samples.

**Table 2:** Intra- and interassay variability of the real-time PCR protocol

Standard dilution	Intra-assay variability		Interassay variability	
	Mean Ct^a^	CV^a^ (%)	Mean Ct^a^	CV^a^ (%)
10^−5^ ng/μl	14.18	0.84	14.51	2.2
10^−6^ ng/μl	17.81	0.23	18.38	3.1
10^−7^ ng/μl	21.18	0.37	21.31	0.7
10^−8^ ng/μl	24.78	0.77	24.90	0.9
10^−9^ ng/μl	27.89	4.0	28.28	1.5

^a^Values represent the mean threshold cycle (Ct) and coefficient of variation (CV) of three replicates in the same (intra-assay) and in three different (inter-assay) qPCR runs

#### eDNA interference in the qPCR counts

**Fig. 1:**
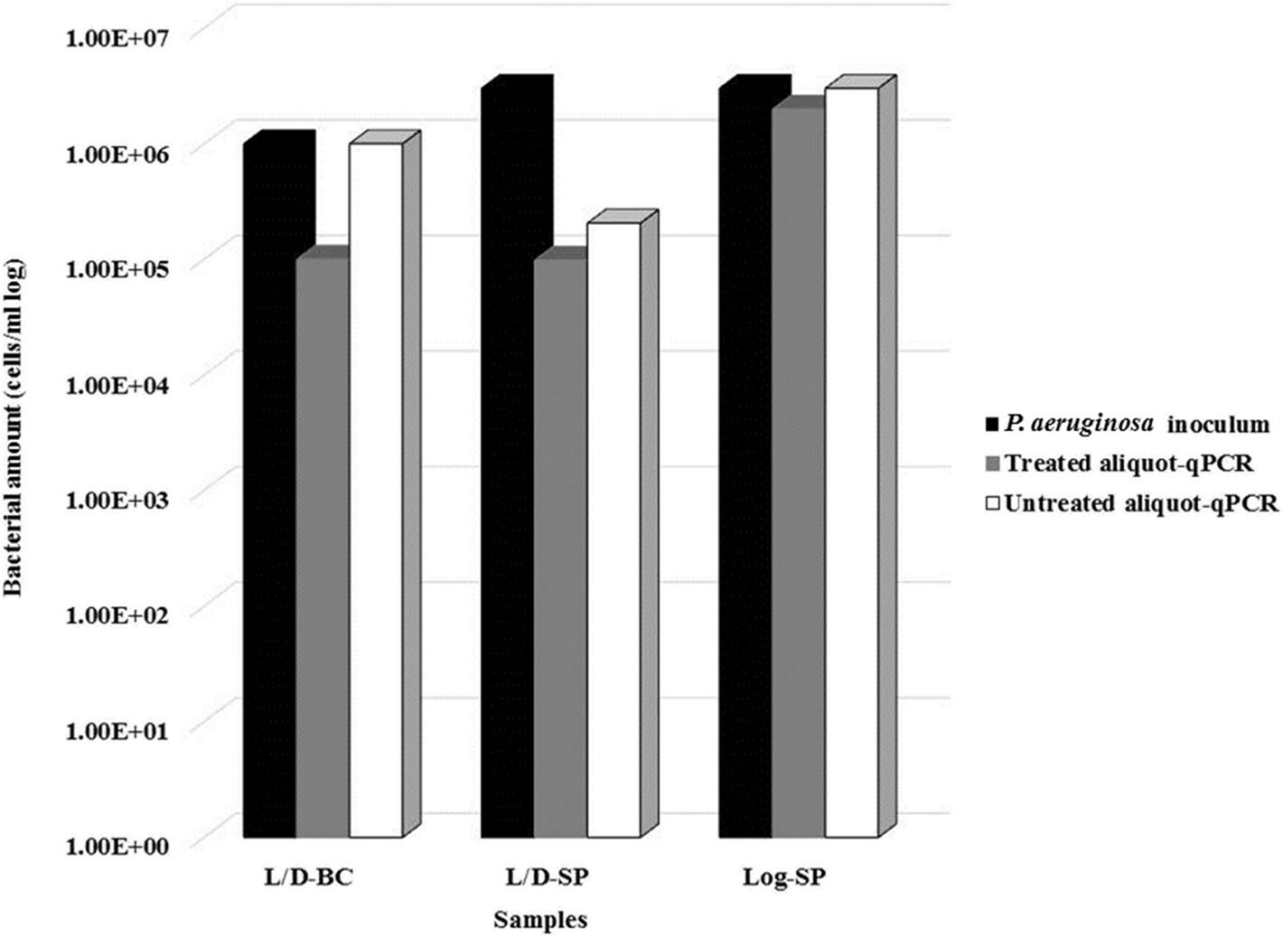
Effect of DNase I treatment on *P. aeruginosa* quantification by qPCR. The effect of DNase I treatment was assessed by comparing the qPCR counts of two aliquots of the same sample, one digested and one undigested. L/D-BC: *P. aeruginosa* ATCC 9027 broth culture containing live and dead cells. L/D-SP: sputum sample spiked with 3 × 10^6^ cells of a broth culture containing live and dead cells. Log-SP: sputum sample spiked with 3 × 10^6^ cells of a log phase culture

The possible interference of eDNA in the qPCR counts was evaluated using 3 types of samples (Fig. 1): a crude extract of a *P. aeruginosa* ATCC 9027 broth culture containing a 1:10 mixture of live (log phase) and dead (heat-killed) cells; a DNA extract from a sputum sample spiked with a mid-log phase culture of *P. aeruginosa* ATCC 9027; and a DNA extract of a sputum sample spiked with a 1:10 mixture of live and dead *P. aeruginosa* ATCC 9027 cells. Each sample was divided into two aliquots, of which one was digested with DNase I and the other was not treated. Although a lower amount of total DNA was consistently recovered from treated compared with untreated aliquots (data not shown), similar qPCR counts were always found in untreated and DNase-treated aliquots of spiked sputum samples; these counts consistently matched the live portion of the whole inoculum. The bacterial counts of DNase-digested aliquots were lower (by 1 log) than those of untreated aliquots only in the crude extract of the *P. aeruginosa* broth culture, suggesting that the commercial kit used for direct DNA extraction from sputum samples efficiently removed the interference due to dead cells or eDNA. When the same analysis was performed in 26 CF sputum samples of clinical origin, no significant (*p* > 0.05) differences in qPCR counts were obtained in both untreated and DNase I-treated aliquots, confirming the absence of eDNA in the direct DNA extract.

### Comparison of culture and qPCR results

Eighty-eight CF sputum samples – 41 CP and 47 CN – were analysed for their *P. aeruginosa* content by the newly-developed qPCR protocol. The results were compared to those obtained by the routine culture method (Table 3). The qPCR results were similar to the CFU/ml counts in 31 CP samples and were higher in 10 (see Additional file 1: Table S1). Moreover, in 9 CN samples qPCR detected *P. aeruginosa* with abundances up to 10^6^ cells/ml (see Additional file 2: Table S2).

**Table 3:** Comparison of qPCR and culture in *P. aeruginosa* detection in 88 CF sputum samples

CF samples	*ecfX*-PCR amplification^a^	qPCR > CFU count
Culture-positive (*n* = 41)	41/41	10/41
Culture-negative (*n* = 47)	9/47	na

^a^the culture-based counts of CP samples were in the following ranges (CFU/ml): 10^5^–10^6^ (*n* = 23), 10^4^–10^5^ (*n* = 9), 10^3^–10^4^ (*n* = 4), and 10^2^–10^3^ (*n* = 5); na, not applicable

qPCR was significantly more efficient (*p* < 0.05) than routine culture in detecting *P. aeruginosa* in CN samples.

### Influence of sample storage at 4 °C on culture and qPCR *P. aeruginosa* counts

**Fig. 2:**
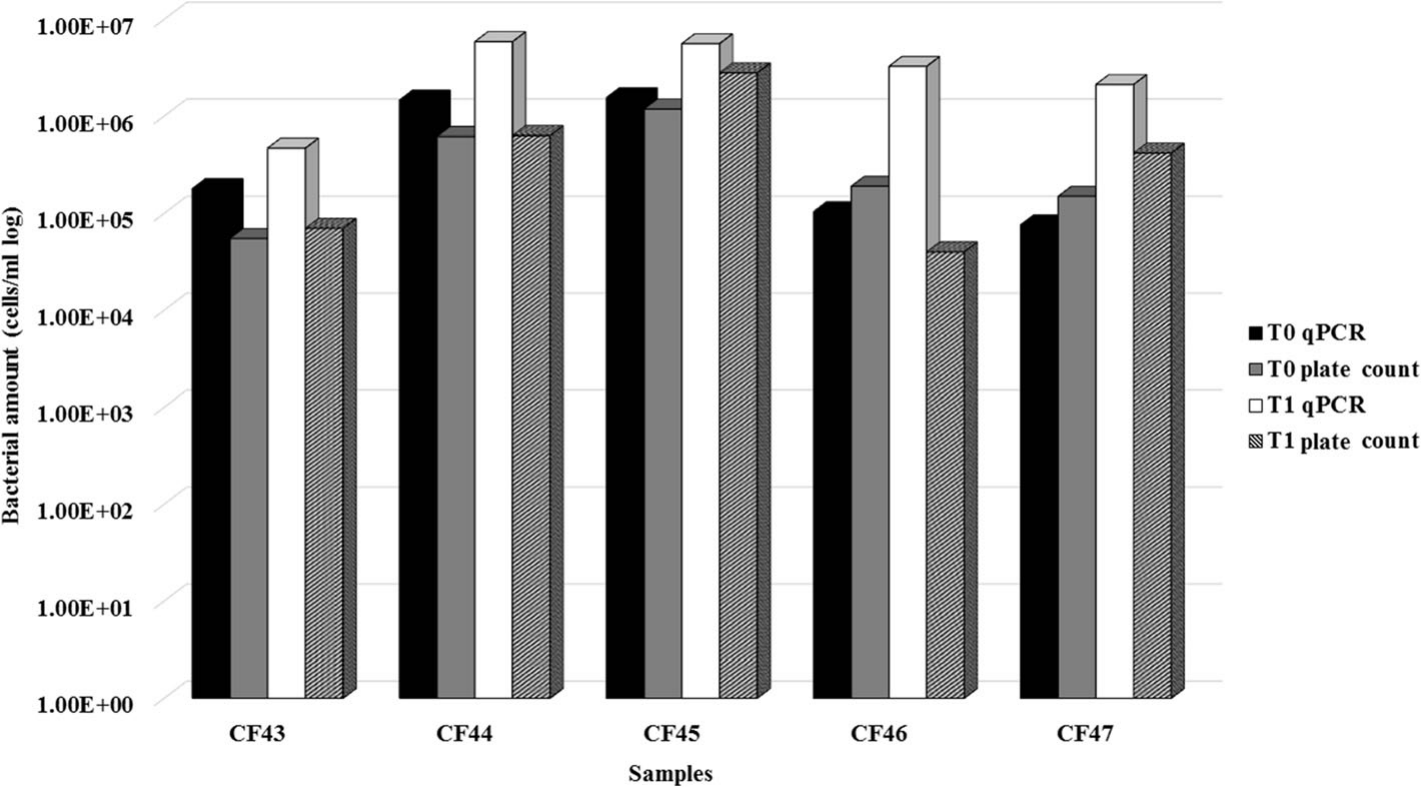
Influence of sample storage at 4 °C on *P. aeruginosa* plate and qPCR counts. Plate and qPCR counts of 5 sputum samples performed on the day of sampling (T0) and after storage at 4 °C for 1 week (T1)

To investigate whether the sputum samples were affected by storage for a week before analysis, we investigated the influence of storage at 4 °C on plate and qPCR *P. aeruginosa* counts of 5 sputum samples (CF43–47). Counts were performed on the day of sampling (T0) and after sample storage at 4 °C for a week (T1). Plate counts results were comparable (< 0.5 log difference) at T0 and T1 for 4 samples (CF43, CF44, CF45, and CF47), whereas the count was slightly lower (T0, 1.04 × 10^5^ vs T1, 4.00 × 10^4^ CFU/ml) at T1 for the fifth (CF46). qPCR counts did not show marked differences (< 0.5 log difference) between T0 and T1 in 3 samples (CF43, CF44 and CF45), whereas in the remaining 2 samples (CF46 and CF47) they were higher at T1 (Fig. 2). Whereas at T0 the qPCR and plate counts did not show clear differences, the qPCR counts were uniformly higher at T1 than at T0 and also than the plate counts, suggesting the induction of non-culturable forms during storage at 4 °C, after initial multiplication.

## Discussion

In CF lung infection, the possible shift of *P. aeruginosa* to a dormant, non-culturable form [4] can hamper pathogen eradication, leading to recurrent episodes of relapse [19]. The urgent need for a reliable routine technique capable of detecting all the different forms of *P. aeruginosa* is thus widely acknowledged. However, none of the protocols devised to date has proved entirely satisfactory, due to suboptimal specificity and sensitivity [20] and to their inability to discriminate between live and dead cells or to exclude the possible interference of eDNA in bacterial counts [11].

In this study, we described a new real-time PCR targeting *ecfX*, which amplifies a 145 bp sequence, and demonstrated that it reliably detected and quantified *P. aeruginosa* in CF sputum samples. Notably, none of the previously described *ecfX*-targeting qPCR protocols [7, 15, 21], used alone, has provided the desired specificity and sensitivity, possibly because they targeted different sequences in the same gene. The SYBR Green *ecfX*-targeting PCR described herein provided several major advantages compared with routine culture, including faster (a few hours) and reliable pathogen detection and a very high sensitivity threshold (70 cells/ml vs. 100 CFU/ml) that was comparable to the one reported for probe-based assays [22]. With regard to DNA purity and abundance, spiking assays performed with live and dead cells after excluding the presence of PCR inhibitors, showed that qPCR counts matched the amount of the live inoculum. Conservation of the target sequence was supported by its detection in all the CP samples analysed and in 96.6% of the *P. aeruginosa* strains isolated from samples of different origins; on the other hand, the 19 *ecfX* copies contained in the *P. aeruginosa* genome make mutational events affecting all of them unlikely. In addition, the absence of cross-reaction with similar bacterial species supports protocol specificity. These data, together with our previous report of real-time PCRs targeting the *P. aeruginosa* genes *gyrB* and *oprL* [23], highlight the efficiency of the qPCR protocol described here, which reliably detected *P. aeruginosa* by targeting a single gene. Experiments are currently under way to achieve further improvements, both by designing new primer pairs inside the 145 bp amplicon and by developing an *ecfX* Taqman probe; sequencing of 23S rDNA amplicons is also being performed to confirm *P. aeruginosa* identification by the newly-developed PCR protocol [24].

eDNA detection is a longstanding limitation in qPCR assays. Treatment of biofilm samples with propidium or ethidium monoazide before DNA extraction [25] has proved unsatisfactory [26]. The DNase I digestion assays performed in the present study provided similar qPCR counts of both digested and undigested aliquots of spiked sputum samples and demonstrated that the counts corresponded with the live bacterial population. These findings, coupled with the results obtained in untreated and DNase-treated aliquots of 26 clinical CF sputum samples, suggest that the DNA extraction procedure from sputum samples efficiently excluded eDNA as well as DNA from dead bacterial cells, which may be too small and/or too damaged to be retained by the DNA purification column.

The discrepancy between the culture-based and the qPCR approach, i.e. *P. aeruginosa* detection in 9/47 CN samples and the greater bacterial abundance found in 10/41 CP samples, supports the ability of the proposed PCR protocol to provide a fast and reliable diagnosis and suggests that VBNC forms of *P. aeruginosa* may be found in CF samples, in line with earlier works [4, 5, 23, 27]. Moreover, the ability of antibiotics to induce a VBNC state in biofilm-growing *Staphylococcus aureus* [28] further supports the notion that a VBNC *P. aeruginosa* population may survive in the CF pulmonary biofilm subjected to extended and repeated antibiotic treatment [19].

## Conclusions

The pivotal role of *P. aeruginosa* lung infections in CF patients stresses the urgent need for efficient methods to diagnose *P. aeruginosa* infection. Compared to routine culture methods, the qPCR-based procedure described herein allows faster and more reliable pathogen detection. Moreover, by detecting all *P. aeruginosa* phenotypes, including dormant forms, it enables monitoring the effectiveness of antibiotic treatment, providing new insight into the actual evolution of the infection.

## Additional files

Additional file 1: **Table S1.** Comparison of plate and qPCR quantification of *P. aeruginosa* in 41 CP samples from CF patients. Description: *P. aeruginosa* abundance in the 41 CP samples quantified by both plate count and qPCR. Higher qPCR than plate counts suggest the presence of non-culturable forms and are indicated by a star. (DOCX 19 kb)

Additional file 2: **Table S2.** qPCR quantification of 9 CN sputum samples from CF patients. Description: *P. aeruginosa* quantification by qPCR in 9 CN sputum samples; the samples which were shown to contain a *P. aeruginosa* amount under the LOD of the developed qPCR were indicated as <LOD. (DOCX 18 kb)

## Abbreviations

CF: Cystic Fibrosis
CN: Culture-Negative
CP: Culture-Positive
Ct: Threshold Cycle
CV: Coefficients of Variation
eDNA: extracellular DNA
LOD: Limit of Detection
PBS: Phosphate Buffered Saline
VBNC: Viable But Non-Culturable
