# Supplementary material for: Detection of viable but non-culturable Pseudomonas aeruginosa in cystic fibrosis by qPCR: a validation study
Source: BMC Infect Dis. 2018 Dec 27;18:701. doi: 10.1186/s12879-018-3612-9 (PMC6307279; doi:10.1186/s12879-018-3612-9)
Supplement: Supplementary file 1 — Table S1. Comparison of plate and qPCR quantification of P. aeruginosa in 41 CP samples from CF patients. Description: P. aeruginosa abundance in the 41 CP samples quantified by both plate count and qPCR. Higher qPCR than plate counts suggest the presence of non-culturable forms and are indicated by a star. (DOCX 19 kb) [file 12879_2018_3612_MOESM1_ESM.docx]

**Additional Table S1. Comparison of plate and qPCR quantification of**

***P. aeruginosa* in 41 CP samples from CF patients**

| **Sample** | **Culture (CFU/ml)** | **qPCR (cells/ml)** |
| --- | --- | --- |
| CF4 | 10^3^-10^4^ | 7.45E+05* |
| CF5 | 10^4^-10^5^ | 1.94E+05 |
| CF6 | 10^4^-10^5^ | 1.27E+04 |
| CF7 | 10^5^-10^6^ | 3.32E+06 |
| CF8 | 10^5^-10^6^ | 5.32E+06* |
| CF9 | 10^2^-10^3^ | 1.90E+03 |
| CF10 | 10^5^-10^6^ | 2.80E+07* |
| CF11 | 10^5^-10^6^ | 7.43E+06* |
| CF20 | 10^4^-10^5^ | 1.00E+06* |
| CF21 | 10^4^-10^5^ | 1.66E+06* |
| CF22 | 10^5^-10^6^ | 1,53E+06 |
| CF23 | 10^4^-10^5^ | 3,50E+04 |
| CF24 | 10^4^-10^5^ | 1,42E+05 |
| CF25 | 10^5^-10^6^ | 3.43E+05 |
| CF26 | 10^5^-10^6^ | 5.95E+06* |
| CF27 | 10^5^-10^6^ | 2.33E+05 |
| CF28 | 10^2^-10^3^ | 3.80E+02 |
| CF29 | 10^3^-10^4^ | 2.01E+03 |
| CF30 | 10^5^-10^6^ | 2.74E+05 |
| CF32 | 10^2^-10^3^ | 1.71E+02 |
| CF34 | 10^5^-10^6^ | 5.94E+05 |
| CF35 | 10^2^-10^3^ | 3.01E+06* |
| CF36 | 10^5^-10^6^ | 2.92E+05 |
| CF37 | 10^3^-10^4^ | 1.24E+05* |
| CF38 | 10^5^-10^6^ | 2.45E+05 |
| CF39 | 10^5^-10^6^ | 6.13E+05 |
| CF40 | 10^4^-10^5^ | 5.15E+06* |
| CF41 | 10^5^-10^6^ | 4.57E+05 |
| CF43 | 10^4^-10^5^ | 1.80E+05 |
| CF44 | 10^5^-10^6^ | 1.46E+06 |
| CF45 | 10^5^-10^6^ | 1.55E+06 |
| CF46 | 10^5^-10^6^ | 1.04E+05 |
| CF47 | 10^5^-10^6^ | 7.06E+04 |
| CF51 | 10^5^-10^6^ | 7.80E+05 |
| CF52 | 10^5^-10^6^ | 4.09E+05 |
| CF64 | 10^4^-10^5^ | 3.30E+05 |
| CF82 | 10^3^-10^4^ | 1.00E+04 |
| CF83 | 10^5^-10^6^ | 4.70E+05 |
| CF84 | 10^2^-10^3^ | 3.20E+02 |
| CF87 | 10^5^-10^6^ | 2.00E+05 |
| CF88 | 10^5^-10^6^ | 8.60E+05 |

*qPCR counts higher (> 0.5 log) than the plate counts
