# Supplementary material for: Detection of viable but non-culturable Pseudomonas aeruginosa in cystic fibrosis by qPCR: a validation study
Source: BMC Infect Dis. 2018 Dec 27;18:701. doi: 10.1186/s12879-018-3612-9 (PMC6307279; doi:10.1186/s12879-018-3612-9)
Supplement: Supplementary file 2 — Table S2. qPCR quantification of 9 CN sputum samples from CF patients. Description: P. aeruginosa quantification by qPCR in 9 CN sputum samples; the samples which were shown to contain a P. aeruginosa amount under the LOD of the developed qPCR were indicated as <LOD. (DOCX 18 kb) [file 12879_2018_3612_MOESM2_ESM.docx]

**Additional Table S2. qPCR quantification of**

***P. aeruginosa* in 9 CN samples from CF patients**

| **Sample** | **qPCR (cells/ml)** |
| --- | --- |
| CF33 | 5.42E+03 |
| CF49 | <LOD* |
| CF54 | 2.80E+06 |
| CF62 | 3.20E+02 |
| CF67 | 1.00E+03 |
| CF68 | 1.80E+02 |
| CF81 | <LOD* |
| CF85 | 1.60E+04 |
| CF86 | 4.00E+03 |

* positive qPCR, bacterial abundance < LOD
